# A pilot clinical study of resveratrol in postmenopausal women with high body mass index: effects on systemic sex steroid hormones

**DOI:** 10.1186/s12967-014-0223-0

**Published:** 2014-08-14

**Authors:** H-H Sherry Chow, Linda L Garland, Brandy M Heckman-Stoddard, Chiu-Hsieh Hsu, Valerie D Butler, Catherine A Cordova, Wade M Chew, Terri L Cornelison

**Affiliations:** University of Arizona Cancer Center, 1515 N Campbell Ave, 85724 Tucson, AZ USA; Division of Cancer Prevention, National Cancer Institute, Bethesda, Maryland

**Keywords:** Resveratrol, Sex steroid hormones, High adiposity, Post-menopausal women

## Abstract

**Background:**

Breast cancer risk is partially determined by several hormone-related factors. Preclinical and clinical studies suggested that resveratrol may modulate these hormonal factors.

**Methods:**

We conducted a pilot study in postmenopausal women with high body mass index (BMI ≥ 25 kg/m^2^) to determine the clinical effect of resveratrol on systemic sex steroid hormones. Forty subjects initiated the resveratrol intervention (1 gm daily for 12 weeks) with six withdrawn early due to adverse events (AEs). Thirty-four subjects completed the intervention.

**Results:**

Resveratrol intervention did not result in significant changes in serum concentrations of estradiol, estrone, and testosterone but led to an average of 10% increase in the concentrations of sex steroid hormone binding globulin (SHBG). Resveratrol intervention resulted in an average of 73% increase in urinary 2-hydroxyestrone (2-OHE_1_) levels leading to a favorable change in urinary 2-OHE_1_/16α-OHE_1_ ratio. One participant had asymptomatic Grade 4 elevation of liver enzymes at the end of study intervention. Two subjects had Grade 3 skin rashes. The remaining adverse events were Grade 1 or 2 events. The most common adverse events were diarrhea and increased total cholesterol, reported in 30% and 27.5% of the subjects, respectively.

**Conclusion:**

We conclude that among overweight and obese postmenopausal women, daily 1 gm dose of resveratrol has favorable effects on estrogen metabolism and SHBG. Further placebo-controlled studies are needed to confirm our findings on these hormone-related breast cancer risk factors and the attribution of the adverse effects observed in the study population.

**Trial registration:**

ClinicalTrials.gov: NCT01370889.

## Background

Breast cancer risk is partially determined by several hormone-related factors, and it has long been hypothesized that high levels of endogenous hormones, especially estrogens, may increase breast cancer risk. After menopause, ovarian production of estrogens has ceased. The circulating estrogens are synthesized in the adipose tissue by enzymatic aromatization of androgenic precursors. Estrogen synthesis in adipose tissue is not regulated by a feedback mechanism and is directly correlated with the amount of adipose tissue. Postmenopausal women with high adiposity produce elevated levels of estrogens, a mechanism thought to link high adiposity with breast cancer [1–5]. In addition, high adiposity is associated with a reduced sex hormone binding globulin (SHBG) in postmenopausal women [1–4] which results in an increase in the fraction of bioavailable sex steroid hormones. The Endogenous Hormones and Breast Cancer Collaborative Group reanalyzed the worldwide data from nine prospective studies to examine the relationship between the levels of endogenous sex hormones and breast cancer risk in postmenopausal women [6]. The risk for breast cancer increased significantly with increasing concentrations of all sex hormones examined: total estradiol, free estradiol, non-SHBG-bound estradiol, estrone, estrone sulfate, and testosterone. SHBG was associated with a decrease in breast cancer risk. In addition, studies have shown that estrogen metabolites vary in estrogenic and genotoxic potential with 16α-hydroxyestrone (16α-OHE_1_) being mitogenic in breast cancer cells whereas 2-hydroxyestrone (2-OHE_1_) is considered an estrogen receptor antagonist [7,8]. Some observation studies have demonstrated a correlation between a low urinary 2-OHE_1_/16α-OHE_1_ ratio and increased breast cancer risk, whereas others have not shown the same correlation [9,10].

Resveratrol, a phytochemical produced by a restricted number of plant species in response to stress, has shown compelling breast cancer preventive activities in preclinical studies. Resveratrol has been shown to suppress proliferation of both ER-positive and negative breast cancer cells in cell culture systems [11,12]. It acts as an estrogen agonist or antagonist depending on the cell types, estrogen receptor isoform, and the presence of endogenous estrogens [13,14]. Some but not all studies showed that resveratrol inhibits aromatase in breast cancer cells [15,16]. In addition, resveratrol has been shown to modulate phase I and phase II enzymes involved in the activation or detoxification of drugs, endogenous hormones and carcinogens in preclinical studies [17–21]. Our prior clinical study showed that resveratrol taken at 1 gm daily for 4 weeks led to inhibition of the activity of cytochrome P450 (CYP) 3A4, 2D6, and 2C9 and induction of CYP1A2 [22]. Because the formation of 2-OHE_1_ and 16α-OHE_1_ was primarily catalyzed by CYP 1A2 and 3A4, respectively, changes in CYP isozyme activities may lead to changes in the balance between mitogenic and protective estrogen metabolites.

Here, we report a pilot study conducted in postmenopausal women with high body mass index (BMI) to determine the modulating effects of resveratrol on circulating sex steroid hormones and estrogen metabolites to evaluate its potentials for breast cancer prevention.

## Methods

### Study design

The study was an open label, single-arm intervention trial in postmenopausal women with high BMI. The primary endpoint of the study was change in serum estradiol levels. Secondary endpoints included estrone, testosterone, SHBG, 2-OHE_1_/16α-OHE_1_ ratio. Safety of resveratrol intervention was assessed by reported adverse events, complete blood count, and comprehensive metabolic panel.

### Study drugs

Resveratrol drug product was supplied by Royalmount Pharma, Inc. through the Division of Cancer Prevention, National Cancer Institute. Resveratrol caplets were manufactured by Pharmascience Inc. using synthetic resveratrol. Resveratrol purity was assessed by HPLC with UV detection. Each study caplet contains 500 mg resveratrol plus inert pharmaceutical excipients. The study caplets were stored at room temperature and protected from environmental extremes.

### Study population

We recruited healthy postmenopausal women with BMI ≥ 25 kg/m^2^ at study entry. Postmenopausal was defined as amenorrhea for at least 12 months, or history of hysterectomy and bilateral salpingo-oophorectomy, or at least 55 years of age with prior hysterectomy with or without oophorectomy, or age 35 to 54 with a prior hysterectomy without oophorectomy or with a status of ovaries unknown with documented follicle-stimulating hormone level demonstrating elevation in postmenopausal range. Participants were required to have normal liver and renal function. Study exclusion criteria included invasive cancers within the past 5 years, within 3 months of or concurrent usage of other investigational agents, a history of allergic reactions attributed to resveratrol, uncontrolled acute or chronic diseases, within 3 months of or concurrent usage of hormonal therapy, selective estrogen-receptor modulators or aromatase inhibitors, regular usage of estrogenic supplements, or concurrent use of anti-diabetic drugs, warfarin or phenytoin. The study was approved by the University of Arizona Human Subjects Protection Program. Written informed consent was obtained from all participants.

### Study procedures

During the initial visit, consented study subjects underwent medical history evaluation and had a fasting blood sample collected for complete blood count and comprehensive metabolic panel. Eligible subjects underwent a minimum of 2 weeks of washout in which they were required to limit resveratrol containing foods and products including wine (red and white), peanuts, mulberries, grapes (seeds, skin, stalks), cranberries, blueberries, huckleberries, or any food containing these ingredients. After the washout period, subjects returned to the clinic for the collection of a fasting blood sample that was used to measure baseline serum hormone levels and study agent levels. Participants were instructed to collect a morning urine void for three consecutive mornings including the morning of the scheduled baseline visit. The urine samples were kept at room temperature before they were brought to the clinic, typically around 48 hours since the initial collection. The urine collection was used for baseline urinary estrogen metabolite analyses. Collected serum and urine samples were stored at −80°C prior to analysis. The storage condition has minimal effects on the analytes of interest.

Following the completion of the baseline sample collection, subjects took 1 gm dose of resveratrol once a day (two 500 mg caplets QD) with food for 12 weeks and continued to limit resveratrol containing foods and products. At week 6, subjects returned to have a blood sample collected for study agent level analysis, return unused pills for a pill count, and review the side effect diary with study staff.

Subjects returned at the end of the 12-week intervention to return unused drugs and review the side effect diary with study staff. A fasting blood sample was collected for clinical labs, post-intervention serum hormone and study agent level analyses. Three morning urine voids were collected for post-intervention urinary estrogen metabolite analyses. Following the resveratrol intervention, study participants were followed for 2 weeks for any adverse reactions.

Safety of resveratrol intervention was assessed by reported adverse events and clinical labs. Adverse events were graded using NCI Common Terminology Criteria for Adverse Events (CTCAE) version 4.0.

Serum estradiol and estrone concentrations were measured by a sensitive and specific liquid chromatography-tandem mass spectrometry assay [23] with minor modifications. Serum testosterone concentrations were measured by a sensitive and specific liquid chromatography-tandem mass spectrometry assay [24] with minor modifications to improve assay specificity. SHBG was measured using an ELISA based immunoassay (GenWay Biotech, Inc.). Urinary 2-OHE_1_ and 16α-OHE_1_ were determined using an ELISA based immunoassay (Immuna Care Corp.).

Plasma resveratrol and metabolite concentrations were determined using a published HPLC method with UV detection [25]. Plasma concentrations of resveratrol metabolites were estimated based on the calibration curve established with resveratrol standard. The identity of resveratrol and its metabolites was confirmed by HPLC in tandem with mass spectrometry by monitoring the parent/product ion transitions of resveratrol and metabolites [25].

### Statistical analysis

Descriptive statistics, e.g. mean and standard deviation, were calculated for each of the endpoints. A two-sided paired t test was performed to test if the percent change from baseline to post-intervention in each of the endpoints is significantly different from zero. Because of the exploratory nature, analyses of these endpoints were not corrected for multiple comparisons. Comparison of resveratrol/metabolite levels between the mid-study and post-interventions visits was achieved by a linear mixed effects model with a random intercept and adjustment for elapsed time from the prior dose. Descriptive statistics was performed on the type and frequency of all adverse events.

## Results

Forty-six subjects were consented between June 2011 and March 2012 with six found to not meet all inclusion criteria. Forty subjects initiated the resveratrol intervention with six withdrawn early due to adverse events. Safety data were analyzed on all subjects who initiated the resveratrol intervention (n = 40). Systemic hormone and estrogen metabolite data were analyzed on subjects who completed 12 weeks of resveratrol intervention (n = 34). Table 1 summarizes the demographics of study subjects who completed the 12-wk intervention. The average age was 58 ± 8 years. The average BMI and body weight was 32.9 ± 6.0 kg/m^2^ and 91.1 ± 18.0 kg, respectively, at baseline and was 32.6 ± 6.3 kg/m^2^ and 90.3 ± 19.6 kg, respectively, post-intervention. Participants took an average of 95% of the assigned pills.

**Table 1:** **Demographic characteristics of study subjects who completed the 12-wk intervention period (n =34)**

Age at Enrollment, yr	58 ± 8^a^	
Race (White/African American/Mixed)	32/1/1	
Ethnicity (Hispanic/Non-Hispanic)	5/29	
BMI, mean ± SD, kg/m^2^		
Baseline	32.9 ± 6.0	
Post-intervention	32.6 ± 6.3	*P* = 0.16^b^
Weight, mean ± SD, kg		
Baseline	91.1 ± 18.0	
Post-intervention	90.3 ± 19.6	*P* = 0.24^b^

^a^Data are presented as mean ± SD.

^b^Derived from a paired t-test for the change from baseline.

Table 2 summarizes the baseline and post-intervention circulating levels of sex steroid hormones and estrogen metabolites. The mean baseline serum concentrations of estradiol, estrone, and testosterone were 12.3 ± 20.7 pg/ml, 26.1 ± 12.7 pg/ml, 0.18 ± 0.11 ng/ml, respectively. Resveratrol intervention did not result in significant changes in these sex steroid hormones. The mean baseline SHBG concentrations were 42.1 ± 17.6 nmol/L. Resveratrol intervention induced an average of 10% increase in serum SHBG concentrations (*p* < 0.01). The mean baseline urinary 2-OHE_1_, 16α-OHE_1_, 2-OHE_1_/16α-OHE_1_ were 9.5 ± 8.8 ng/mg creatinine, 6.6 ± 6.1 ng/mg creatinine, and 1.7 ± 1.4, respectively. Resveratrol intervention resulted in a 73.2% increase in urinary 2-hydroxyestrone (2-OHE_1_) levels (*p* < 0.01), leading to an 84.5% increase in urinary 2-OHE_1_/16α-OHE_1_ ratio (*p* < 0.01).

**Table 2:** **Summary of baseline and post-intervention systemic sex steroid hormones and estrogen metabolites (n = 34)**

**Sex steroid hormones**	**Baseline**	**Post-intervention**	**% change**	***P*** ^***b***^
Estradiol, pg/ml	12.3 ± 20.7^a^	15.5 ± 34.9	22.4 ± 178	0.47
Estrone, pg/ml	26.1 ± 12.7	25.8 ± 14.3	1.4 ± 38.3	0.83
Testosterone, ng/ml	0.18 ± 0.11	0.18 ± 0.12	−0.85 ± 27.2	0.86
SHBG, nmol/L	42.1 ± 17.6	45.3 ± 18.0	10.0 ± 17.8	<0.01
2-OHE_1_, ng/mg creatinine	9.5 ± 8.8	13.8 ± 8.75	73.2 ± 132	<0.01
16α-OHE_1_, ng/mg creatinine	6.6 ± 6.1	6.6 ± 3.0	15.9 ± 51.0	0.08
2-OHE_1_/16α-OHE_1_ ratio	1.7 ± 1.4	2.7 ± 2.7	84.5 ± 175	<0.01

^a^Data are presented as mean ± SD.

^b^Derived from a paired t-test for % change from baseline to post-intervention.

**Figure 1:**
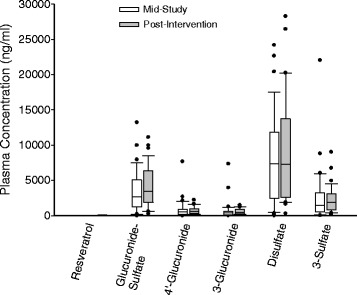
**Resveratrol and metabolites concentrations in the plasma samples collected at mid-study and post-intervention visits.** Plots illustrate the median, 25th, and 75th percentiles as vertical boxes with 10th and 90th percentiles as error bars.

Resveratrol and metabolites were not detectable in any of the baseline plasma samples. Resveratrol and metabolites concentrations in the plasma samples collected at mid-study and post-intervention visits are shown in Figure 1. The average elapsed time since the prior dose was 965 ± 381 and 818 ± 217 minutes, respectively, for the mid-study and post-intervention samples. Resveratrol was not detected (assay detection limit of 80 pg/ml) in these samples while the resveratrol sulfate and/or glucuronide conjugates were present at high concentrations. Levels of the resveratrol metabolites were similar in mid-study and post intervention samples.

Table 3 summarizes AEs observed in study participants after initiation of the resveratrol intervention. One participant had asymptomatic Grade 4 elevation in hepatic ALT and AST enzymes at the end of 3-month agent intervention. The participant had a normal hepatitis panel as part of the evaluation of this liver test abnormality and returned every 2–3 weeks for follow-up blood tests for the hepatic panel. The elevated hepatic enzymes returned to normal after less than 3 months of follow-up. Six subjects withdrew early from the resveratrol intervention due to adverse events; two after 12 and 33 days, respectively, due to Grade 3 skin rash, one after 66 days due to Grade 2 diarrhea, one after 10 days due to Grade 2 allergic reaction, one after 39 days due to Grade 2 constipation, and one after one dose due to Grade 2 diarrhea and Grade 1 shortness of breath and wheezing. The remaining AEs were transient and were Grade 1 or 2 events. The most common adverse events were diarrhea and dyslipidemia. Diarrhea was reported in 12 subjects (30%). Increased total cholesterol was observed in 11 subjects (27.5%) with increased LDL, VLDL, and cholesterol/HDL ratio in 7 (17.5%), 4 (10%), 3 (7.5%) subjects, , respectively. Increased triglycerides were observed in 6 (15%) subjects.

**Table 3:** **Summary of adverse events observed in study participants after initiation of the resveratrol intervention (n = 40)**

	**Grade 1** ^**a**^	**Grade 2**	**Grade 3**	**Grade 4**
**Blood disorders**				
Anemia	1^b^	0	0	0
**Ear disorders**				
Tinnitus	1	0	0	0
**Eye disorders**				
Dry eye	1	0	0	0
**Gastrointestinal disorders**				
Diarrhea	6	6	0	0
Dyspepsia	2	1	0	0
Constipation	2	1	0	0
Food poisoning	0	3	0	0
Flatulence	2	0	0	0
Abdominal pain	0	1	0	0
Gastroesophageal reflux	0	1	0	0
Nausea	0	1	0	0
Vomiting	0	1	0	0
Stomach pain	0	1	0	0
Intestinal distress	1	0	0	0
**General disorders**				
Flu-like symptoms	0	3	0	0
Irritability	1	0	0	0
**Immune system disorders**				
Allergic reaction	0	1	0	0
Allergic rhinitis	0	1	0	0
**Infections**				
Upper respiratory infection	0	5	0	0
Sinusitis	0	3	0	0
Urinary tract infection	0	1	0	0
Cold sore on lip	1	1	0	0
Bronchial infection	0	1	0	0
Cold sore in mouth	0	1	0	0
**Investigations**				
↑ Total cholesterol	10	1	0	0
↑ LDL	7	0	0	0
↑ Triglycerides	4	2	0	0
↑ VLDL	4	0	0	0
↑ Cholesterol/HDL ratio	3	0	0	0
↓ HDL	2	0	0	0
↑ non-HDL	1	0	0	0
↑Alanine aminotransferase	2	0	0	1
↑Aspartate aminotransferase	1	0	0	1
**Metabolism and nutrition disorders**				
↑ Fasting glucose	4	0	0	0
Appetite change	2	0	0	0
Hypokalemia	1	0	0	0
**Musculoskeletal and connective tissue disorders**				
Myalgia	0	1	0	0
Back pain	0	1	0	0
Neck pain	0	1	0	0
Arthralgia	0	1	0	0
Swollen knee	0	1	0	0
Body aches	1	0	0	0
Leg pain	1	0	0	0
**Neoplasms**				
Ganglion cyst	1	0	0	0
**Nervous system disorders**				
Headache	4	3	0	0
Vivid dreams	2	0	0	0
Dysesthesia	1	0	0	0
Dysgeusia	1	0	0	0
Weird dreams	1	0	0	0
**Psychiatric disorders**				
Insomnia	1	1	0	0
**Renal and urinary disorders**				
Urinary incontinence	0	1	0	0
Bladder spasm	1	0	0	0
Urinary frequency	1	0	0	0
**Reproductive system disorders**				
Pelvic pain	1	0	0	0
**Respiratory, thoracic and mediastinal disorders**				
Dyspnea	2	0	0	0
Laryngitis	0	1	0	0
Sore throat	0	1	0	0
Wheezing	1	0	0	0
**Skin and subcutaneous tissue disorders**				
Dry skin	3	0	0	0
Rash, maculo-papular	0	0	2	0
Rash, other	1	0	0	0
**Vascular disorders**				
Hot flashes	3	1	0	0

^a^Graded using CTCAE version 4.0.

^b^Number of subjects experienced the event.

## Discussion

Overweight and obesity are associated with an increased risk for postmenopausal breast cancer and poor disease outcome (reviewed by [26,27]). The increased risk is partially determined by several hormone-related factors. To the best of our knowledge, our study is the first to report the clinical activity of resveratrol on circulating sex steroid hormones and estrogen metabolites. The baseline levels of sex steroid hormones and estrogen metabolites were similar to those reported in postmenopausal women in a similar BMI range [4,9,10]. We showed that 1 gm of resveratrol daily for 12 weeks did not alter the serum estrogen and testosterone concentrations in postmenopausal women with high adiposity but significantly increased the concentrations of SHBG, which has been inversely associated to breast cancer risk [6]. We did not observe any significant changes in serum insulin levels (data not shown) and body weight, two major factors known to influence the blood levels of SHBG [28]. Future randomized, controlled trials are needed to confirm the effect of resveratrol on SHBG. Because SHBG is the main transport binding protein for circulating sex steroid hormones, it has been suggested that elevation in SHBG could lead to a decrease in bioavailable sex steroid hormones and their associated bioactivity. We calculated the hormone fractions based on the law of mass action [29] and found that resveratrol intervention did not result in significant changes in bioavailable estradiol but decreased the levels of bioavailable testosterone (from 0.087 ± 0.066 to 0.076 ± 0.050 ng/ml, *p* = 0.03). Further studies are needed to determine the effect of resveratrol-induced hormonal changes on breast cancer risk modulation.

Interestingly, our study showed that 1 gm of resveratrol daily for 12 weeks resulted in a significant increase in urinary 2-OHE_1_ levels leading to a favorable change in urinary 2-OHE_1_/16α-OHE_1_ ratio. The formation of 2-OHE_1_ and 16α-OHE_1_ was primarily catalyzed by cytochrome P450 (CYP) 1A2 and 3A4, respectively. The observed changes in estrogen metabolism is consistent with our prior clinical study that showed an induction of 1A2 and inhibition of 3A4 activity after 4 weeks of 1 gm daily resveratrol dosing [22]. Prior studies have shown that putative cancer preventive compounds derived from cruciferous vegetables, indole-3-carbinol and 3,3′-diindolylmethane, also increased the urinary 2-OHE_1_/16α-OHE_1_ ratio [30–32]. Some observation studies have demonstrated a correlation between a low urinary 2-OHE_1_/16α-OHE_1_ ratio and increased breast cancer risk, whereas others have not shown the same correlation [9,10]. Further research is needed to evaluate whether resveratrol-induced changes in estrogen metabolism would contribute to breast cancer risk modulation.

The resveratrol dose and product used in our study has previously been shown to be safe and well tolerated up to 4 weeks in healthy adults [22]. In this study of overweight or obese postmenopausal women, six subjects withdrew early after taking resveratrol for 1–66 days due to adverse events including diarrhea, constipation, skin rash, allergic reaction, and shortness of breath. One participant had asymptomatic Grade 4 ALT and AST elevation at the post-intervention visit which normalized after less than 3 months of follow-up and was considered possibly related to resveratrol intervention. The common reported adverse events were diarrhea and dyslipidemia. Diarrhea has been reported in previous clinical studies of resveratrol [22,33]. It is not known whether the unfavorable change in the lipid profile is related to the resveratrol intervention in the study population. A recent study showed that 1, 1.5, or 2 g of resveratrol daily for 4 weeks did not change the fasting lipid profile in older overweight/obese adults with impaired glucose tolerance [34] whereas another study showed that supplementation of 1 g resveratrol daily for 45 days improved the HDL levels in diabetic patients [35].

Our study has a number of limitations. First, we have designed this study as a pilot project to assess changes in systemic sex steroid hormones before proceeding to larger trials. Further clinical investigation should include a control arm to minimize potential confounders (such as changes in diet and physical activity) in single arm studies. In addition, we have observed a large inter-individual variation in serum estradiol and estrone levels. Larger sample sizes may be needed to observe a significant effect from the supplementation on these measurements. In addition, future studies should consider measurements of serum hormone levels (such as follicle-stimulating hormone and luteinizing hormone) to confirm postmenopausal status for study entry.

## Conclusions

We conclude that in postmenopausal women with high BMI, daily 1 gm dose of resveratrol had favorable effects on SHBG and estrogen metabolites. Further placebo-controlled studies are needed to confirm our findings on these hormone-related breast cancer risk factors and the attribution of the adverse effects observed in the study population.

## Abbreviations

BMI: Body mass index
AE: Adverse events
SHBG: Sex hormone binding globulin
2-OHE_1_: 2-hydroxyestrone
16α-OHE_1_: 16α-hydroxyestrone
CYP: Cytochrome P450
